# Molecular characterization of human coronaviruses and their circulation dynamics in Kenya, 2009–2012

**DOI:** 10.1186/s12985-016-0474-x

**Published:** 2016-02-01

**Authors:** Lenata A. Sipulwa, Juliette R. Ongus, Rodney L. Coldren, Wallace D. Bulimo

**Affiliations:** College of Health Sciences (COHES), Jomo Kenyatta University of Agriculture and Technology, (JKUAT), Nairobi, Kenya; Department of Emerging Infectious Diseases, US Army Medical Research Unit–Kenya, P.O. Box 606 00621, Village Market, Nairobi, Kenya; Special Foreign Activity of the Walter Reed Army Institute of Research, Silver Spring, MD USA; Department of Biochemistry, School of Medicine, University of Nairobi, Nairobi, Kenya

**Keywords:** Molecular characterization, Human coronaviruses, Prevalence, Kenya, HCOV-NL63, HCoV-OC43, HCoV-229E, HCoV-HKU1

## Abstract

**Background:**

Human Coronaviruses (HCoV) are a common cause of respiratory illnesses and are responsible for considerable morbidity and hospitalization across all age groups especially in individuals with compromised immunity. There are six known species of HCoV: HCoV-229E, HCoV-NL63, HCoV-HKU1, HCoV-OC43, MERS-CoV and SARS-HCoV. Although studies have shown evidence of global distribution of HCoVs, there is limited information on their presence and distribution in Kenya.

**Methods:**

HCoV strains that circulated in Kenya were retrospectively diagnosed and molecularly characterized. A total of 417 nasopharyngeal specimens obtained between January 2009 and December 2012 from around Kenya were analyzed by a real time RT-PCR using HCoV-specific primers. HCoV-positive specimens were subsequently inoculated onto monolayers of LL-CMK2 cells. The isolated viruses were characterized by RT-PCR amplification and sequencing of the partial polymerase (*po*l) gene.

**Results:**

The prevalence of HCoV infection was as follows: out of the 417 specimens, 35 (8.4 %) were positive for HCoV, comprising 10 (2.4 %) HCoV-NL63, 12 (2.9 %) HCoV-OC43, 9 (2.1 %) HCoV-HKU1, and 4 (1 %) HCoV-229E. The Kenyan HCoV strains displayed high sequence homology to the prototypes and contemporaneous strains. Evolution analysis showed that the Kenyan HCoV-OC43 and HCoV-NL63 isolates were under purifying selection. Phylogenetic evolutionary analyses confirmed the identities of three HCoV-HKU1, five HCoV-NL63, eight HCoV-OC43 and three HCoV-229E.

**Conclusions:**

There were yearly variations in the prevalence and circulation patterns of individual HCoVs in Kenya. This paper reports on the first molecular characterization of human Coronaviruses in Kenya, which play an important role in causing acute respiratory infections among children.

## Background

Coronaviruses are enveloped viruses with a linear, non-segmented, positive-sense, single-stranded RNA genome of about 27–32 kb [1]. They are round and sometimes pleiomorphic virions of approximately 80-120 nm in diameter. Their genome is the largest of all RNA viruses [2, 3]. Coronaviruses share features of genome organization and replication strategy, but have different virion morphology and genome lengths. The 20 kb polymerase (*pol*) gene, which covers the 5′ two-thirds of the coronavirus genome, contains two large open reading frames (ORFs), ORF 1a and ORF 1b [1]. ORF 1b hosts the most highly conserved genomic sequences, encoding conserved functions such as polymerase and helicase activity [4]. These functions combined with similarities in replication and expression strategies, exhibit an evolutionary link among coronaviruses, arteriviruses, and toroviruses, thus forming the rationale for placing these viruses in the order *Nidovirales* [1]. Therefore, the highly conserved structure and function of viral polymerases make the *pol* a logical region for making phylogenetic comparisons.

Coronaviruses cause various diseases in different hosts, such as respiratory tract infections, gastroenteritis, hepatitis, and encephalomyelitis in birds and mammals [5]. Coronaviruses are classified into four distinct phylogenetic groups. Whereas alphacoronaviruses, betacoronaviruses and gammacoronaviruses infect mammals, deltacoronaviruses infect avian species [6]. All known human coronaviruses (HCoV) belong to the alphacoronavirus and betacoronaviruses. HCoV cause 30 % of common colds as well as severe infections in infants, children, and immunocompromised persons and the elderly [7]. HCoVs have a worldwide circulation, with reports published in populations of different countries such as Saudi Arabia [8], France [9], Kenya [10] and China [11] among others. However in 2012, a novel betacoronavirus Middle East Respiratory Syndrome Coronavirus (MERS-CoV) was isolated in Jeddah from a patient who was suffering from pneumonia and later died [8]. This MERS-CoV continues to cause fatal severe lower respiratory tract illnesses in various parts of the world, especially in the Middle East. As of February 2015, the World Health Organization had reported 971 laboratory confirmed cases with 356 deaths [12]. In light of the finding of this previously unknown coronavirus, it is probable that coronaviruses, other than those recognized to date, may be circulating in human populations in Kenya. As human coronaviruses are not routinely analyzed in Kenya, this study sought to identify and characterize HCoV strains circulating in different parts of Kenya and thus provide a knowledge base for epidemiological importance of human coronaviruses.

## Methods

### Study sites, inclusion criteria, clinical parameters

Nasopharyngeal (NP) specimens were retrieved from archives of the respiratory virus surveillance program in the Department of Emerging Infectious Diseases (DEID) of the United States Army Medical Research Unit-Kenya (USAMRU-K) [13]. These approximately 10000 archived NP specimens were collected from a study population comprising persons from two months of age onwards who attended outpatient clinics in the hospitals involved in the USAMRU-K respiratory virus surveillance; presenting with influenza like illnesses symptoms. These symptoms were: cough, sore throat, and fever (≥38 °C) within 72 h after onset of illness. The nasoopharyngeal swabs were suspended in virus transport medium (VTM) and stored at −80 °C. These samples had been screened for other respiratory viruses including influenza viruses, HAdV, RSV, HMPV, HSV1, rhinoviruses and non-polio enteroviruses including coxsackie viruses [14].

The surveillance network comprised Mbagathi, Isiolo, Alupe, Kericho, Kisi, Malindi, and Port Reitz district hospitals, as well as the New Nyanza General provincial hospital [13]. These hospitals represent disparate geographic regions and population demographics across the country and were delineated into Central, Northern, Western, Highlands and Coastal regions of Kenya, respectively. Inclusion criteria consisted of being an outpatient aged at least 2 months and having influenza-like-illness (ILI) symptoms [13]. Clinical parameters and symptoms including recent history of ILI, cough, sore throat, difficulty in breathing, chills, nasal stuffiness, runny nose, sputum production, headache, joint pain, fatigue, diarrhea, vomiting and bleeding were documented. Only those samples collected from January 2009 to December 2012 for which specific consent was granted for future use were analyzed. The minimum sample size was 173 as determined by Fischer’s formula [15]. Based on available resources, the final number of samples was determined by using the randomized sampling technique. This involved systematic sampling, where the first sample was picked randomly and thereafter the k^th^ sample selected from a list of samples for each site.

### Ethical approval statement

The Walter Reed Army Institute of Research (WRAIR) Institutional Review Board (IRB) and the Kenya Medical Research Institute (KEMRI) Scientific and Ethics Review Unit (SERU) reviewed and approved this work under protocol numbers WRAIR# 2075 and KEMRI SSC#2673 respectively.

### Molecular screening of the virus isolates

RNA was extracted from 140 μl of nasopharyngeal (NP) specimens using the QIAmp Viral RNA Mini Kit (Qiagen, Inc.,USA) according to the manufacturer’s specifications. Viral RNA was used as a template for real time RT-PCR using HCoV-specific primers as previously described by Kuypers et al. [16]. For MERS-CoV detection, samples were analysed using NCV-2012rRT-PCR assay according to the CDC protocols. Control RNAs used were obtained from ATCC (VR-1558D for OC43 and VR740D for 229E) as well as NL63 RNA from the KEMRI-Welcome Trust research program. The MERS-CoV control RNA came along with the test kit from CDC. Samples that tested positive by real time RT-PCR were inoculated onto monolayers of LL-CMK2 cells (ATCC strain CCL-7) in culture tubes (Nunc, Denmark) to contain 100 μl of clinical specimen. The culture medium consisted of Dulbecco’s minimum essential medium (DMEM, Life Technologies, NY, USA) supplemented with 0.04 μg/ml gentamycin, 100U/mL of penicillin & 100 μg/mL streptomycin (Sigma-Aldrich Co. MO, USA). These were incubated at 33 °C under 5 % CO_2_, and virus growth was monitored for cytopathic effects (CPEs) for up to 10 days. Culture controls were obtained from ATCC and included betacoronavirus 1 (ATCC® VR-1558™) and human coronavirus 229E (ATCC® VR-740™).

### Amplification and nucleotide sequencing of RdRp (pol) gene

A 440 bp portion of the intergenic region of ORF1a and ORF1b of the RNA-dependent RNA polymerase (pol) gene of HCoVs was amplified by RT-PCR following the method described by Woo et al. [17] . RT was performed using the SuperScript III kit (Invitrogen, San Diego, USA) following manufacturer’s instructions. The forward primer was 5′-GGTTGGGACTATCCTAAGTGTGA-3′ whereas the reverse primer was 5′ -CCATCATCAGATAGAATCATCATA-3′. The PCR mixture (50 μl) contained RNA (300 ng), PCR buffer containing 10 mM Tris–HCl, pH 8.3, 50 mM KCl, 3 mM MgCl_2_, 0.01 % gelatin, 200 μM each of deoxynucleoside triphosphates, and 1.0 U of Taq polymerase (Boehringer, Mannheim, Germany). The amplification was carried out as described by Woo et al. [17]. using the ABI 9700 automated thermal cycler (Applied Biosystems, Foster city, USA). The PCR amplicons were purified using Exonuclease I/Shrimp Alkaline Phosphatase (ExoSap-IT) enzyme (Affymetrix, California, USA) and sequenced directly on both strands with the same primers used in the PCR on an automated ABI 3500XL Genetic Analyzer (Applied Biosystems, Foster city, USA). Cycle sequencing was performed using the Big Dye Terminator v3.1 sequencing kit (Applied Biosystems, Foster city, USA) according to manufacturer’s instructions.

### Sequence analysis

The nucleotide sequence fragments were edited and assembled into consensus contigs using DNA baser version 3.2 [18]. The nucleotide sequences were compared against known sequences of coronavirus pol genes in the GenBank database using the BLAST [19]. Prototype nucleotide sequences and sequences showing highest sequence homologies were retrieved from GenBank for further analyses. Multiple sequence alignment of these was performed using Muscle v3.8 software [20]. Phylogenetic relationships were inferred using MrBayes software v3.2 [21] and the tree visualized using Fig Tree v1.4.0 software [22]. Nucleotide sequences of the partial pol genes of the Kenyan HCoV isolates reported in this study are available in GenBank under accession numbers:[GenBank: KP112147 to KP112161].

## Results

A total of 417 nasopharyngeal samples were screened. Among these, 35 tested positive for human coronaviruses, displaying Ct values ranging from 21 to 39. Upon molecular typing the 35 positives, 14 (37 %) belonged to alphacoronaviruses and 21 (63 %) were betacoronaviruses. Amongst the alphacoronaviruses, 4 were HCoV-229E and 10 were HCoV-NL63. Betacoronaviruses consisted of 12 HCoV-OC43 and 9 HCoV-HKU1 species. Thus the prevalence of the various HCoVs was 1 % for 229E, 2 % for NL63, 3 % OC43 and 2 % for HKU1.

**Fig. 1:**
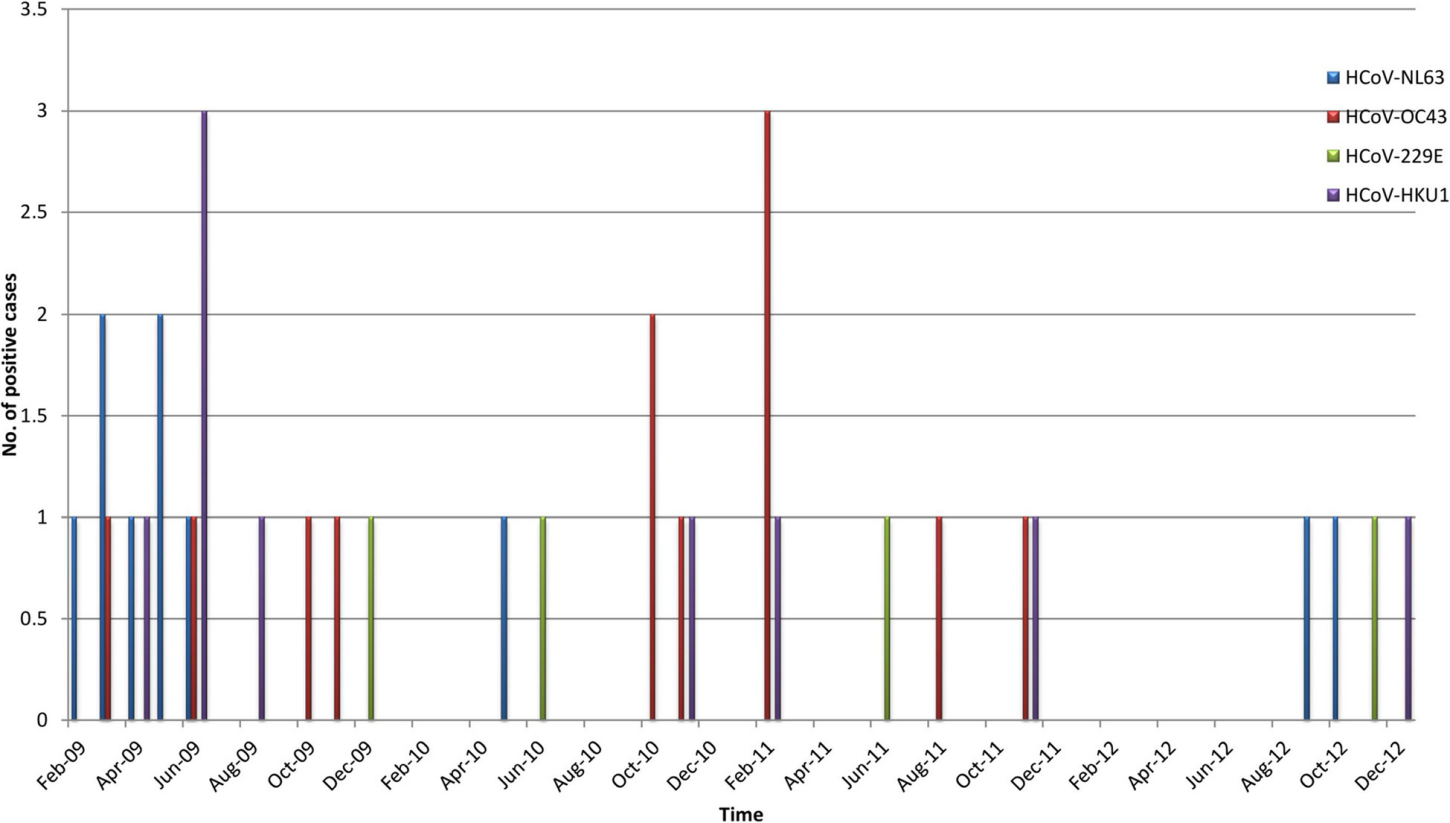
Temporal distribution of HCoVs in Kenya between 2009 to 2012

**Fig. 2:**
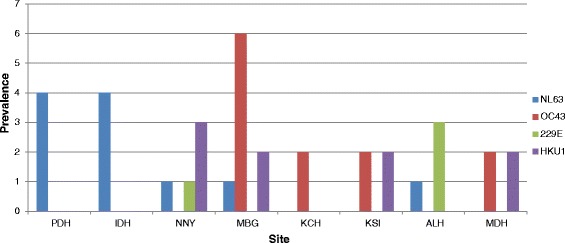
Spatial Distribution of HCoVs in Kenya between 2009 and 2012. Mbagathi, Isiolo, Alupe, Kericho, Kisii, Malindi, and Port Reitz District Hospitals are abbreviated as MBG, IDH, ALH, KCH, KSI, MDH and PDH respectively. New Nyanza General provincial hospital is abbreviated as NNY

HCoV-OC43 was detected in most months in 2009–2011, but was not detected in 2012. The peak circulation was observed in February 2011 (Fig. 1) affecting patients aged 5 months to 5 years. It was most commonly detected in Central Kenya but never in Northern Kenya (Fig. 2). HCoV-NL63 was detected mostly from samples obtained in Northern and Western Kenya and amongst patients aged 3 months to 1.75 years (Fig. 1). HCoV-NL63 predominated from February to June 2009, then disappeared and resurfaced in September 2012.

HCoV-HKU1 was detected all over Kenya, but mostly in the warmer Western and Coastal regions, with peaks observed in June 2009, November 2010, February 2011 and December 2012 (Figs. 1 and 2). It was detected in patients aged 4 months to 6 years. HCoV 229E was only detected in the Western Kenya, from patients aged 9 months to 2 years with peaks detected in July and December (Fig. 2).

The range of ages of the patients in the study was between 2 months and 67 years, with a mean age of 1.5 years and median of 1.67 years. Overall, 94 % of the HCoV were from patients aged <5 years and; 4555 % of the patients were female and 55 % were male. All the patients from whom HCoVs were detected presented with a cough and runny nose, with 50 % of the patients reporting nasal stuffiness. We observed five co-infections with a HCoV. These included two triple co-infection involving HKU1 with influenza A (H3N2) & human adenovirus and NL63 with influenza A (H3N2) & human adenovirus. Three double co-infections were observed. They included HKU1 with RSV, OC43 with influenza A (H1N1)pdm and OC43 with influenza B (Brisbane-like). All the co-infections, except that consisting of HKU1 & RSV presented with vomiting as a symptom. One patient with HCoV-NL63 presented with neurological symptoms. Amongst the patients positive for HCoV-OC43, 90 % presented with vomiting and malaise.

Characterization by cell culture showed that of the 35 positive samples, 12 (34 %) produced cytopathic effects (CPE) in cell culture after incubation for 10 days. PCR amplification of a 440 bp fragment using CoV-specific primers confirmed that the CPE was due to HCoVs. To determine whether there were any HCoVs in the supernatants of the 23 cultures that did not yield CPE, RT-PCR using HCoV-specific primers generated the expected 440 bp amplicon from 3 of these samples.

Homology analyses of the partial nucleotide sequences of the RdRp (*pol*) gene of the Kenyan HCoV against the prototype strains ranged from 59 to 100 % (Table 1). Nucleotide sequence homology within groups ranged from 99 to 100 % for HCoV-OC43, 97–99 % for HCoV-HKU1 and 98–99 % for HCoV-NL63. The Kenya HCoV-229E species had 99 % nucleotide sequence identity compared to the prototype strain.

**Table 1:** Nucleotide sequence homology of the Kenyan HCoV isolates

	NC_005147_OC43	NC_006577_HKU1	NC_002645_229E	NC_005831_NL63	HCoV_016_2010	HCoV_018_2010	HCoV017_2010	HCoV_019_2010	HCoV_014_2010	HCoV_012_2010	HCoV_007_2010	HCoV_013_2010	HCoV_001_2010	HCoV_010_2009	HCoV_008_2009	HCoV_002_2012	HCoV_006_2009	HCoV_009_2010	HCoV_005_2009	HCoV_011_2009	HCoV_015_2009	HCoV_004_2012	HCoV_003_2009
NC_005147_OC43																							
NC_006577_HKU1	81																						
NC_002645_229E	59	62																					
NC_005831_NL63	60	61	75																				
HCoV_016_2010	100	81	59	60																			
HCoV_018_2010	99	82	59	60	99																		
HCoV_017_2010	99	82	59	60	99	100																	
HCoV_019_2010	99	82	59	60	99	100	100																
HCoV_14_2010	99	82	59	60	99	100	100	100															
HCoV_012_2010	99	82	59	60	99	100	100	100	100														
HCoV_007_2010	99	82	59	60	99	100	100	100	100	100													
HCoV_013_2010	99	82	59	60	99	100	100	100	100	100	100												
HCoV_001_2010	80	97	61	60	80	81	81	81	81	81	81	81											
HCoV_010_2009	81	99	63	61	81	82	82	82	82	82	82	82	96										
HCoV_008_2009	81	99	62	61	81	81	81	81	81	81	81	81	97	99									
HCoV_002_2012	59	62	99	75	59	59	59	59	59	59	59	59	62	63	63								
HCoV_006_2009	59	62	99	75	59	59	59	59	59	59	59	59	62	63	63	100							
HCoV_009_2010	59	62	99	75	59	59	59	59	59	59	59	59	62	63	63	100	100						
HCoV_005_2009	60	60	76	99	60	60	60	60	60	60	60	60	60	61	61	76	76	76					
HCoV_011_2009	59	60	75	98	59	60	60	60	60	60	60	60	59	60	60	75	75	75	99				
HCoV_015_2009	60	60	76	99	60	60	60	60	60	60	60	60	60	61	61	76	76	76	100	99			
HCoV_004_2012	60	60	76	99	60	60	60	60	60	60	60	60	60	61	61	76	76	76	100	99	100		
HCoV_003_2009	60	60	76	99	60	60	60	60	60	60	60	60	60	61	61	76	76	76	100	99	100	100	

The numbers represent % nucleotide identities among the strains

**Fig. 3:**
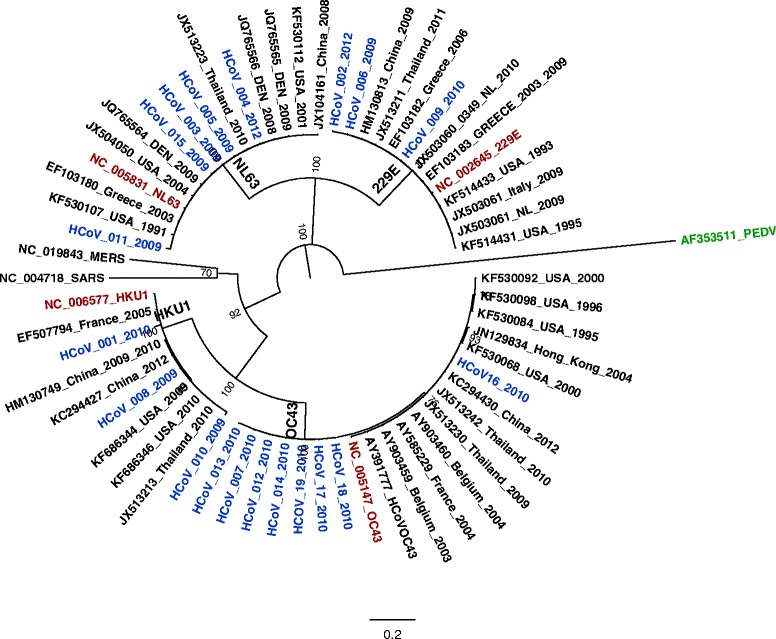
Phylogenetic relationships of partial sequences from the HCoV *pol* gene of the Kenyan isolates with representatives from different species. The Kenyan HCoV sequences are in blue color, the prototype strains are in red and representative strains in black; each sequence is represented by the GenBank accession number. The PEDV in green was used as an out-group. The tree was constructed by Mr. Bayes v3.2, using the general time-reversible nucleotide substitution model. Posterior probability support values are shown as percentages at the nodes

Phylogenetic analyses of the Kenyan isolates based on the nucleotide sequences with representative reference strains revealed separation of the viruses into the two α and β corona-virus groups with further resolution into four species, corresponding to HCoV-OC43, HCoV-NL63, HCoV-229E and HCoV-HKU1 (Fig. 3). The majority (42 %) of the Kenyan isolates clustered within the HCoV-OC43 group, followed by HCoV-NL63 (26 %) and HCoV-HKUI & HCoV-229E (16 % each). All the Kenyan HCoV-OC43 isolates formed a cluster on their own except isolate HCOV-016 which grouped with the other HCoV-OC43 viruses from other countries across the globe. The Kenyan HCoV-NL63 isolates clustered with other HCoV-NL63 viruses. The Kenyan HCoV-HKU1 isolates grouped close to the French virus whose accession number is EF507794 and the Kenyan HCoV-229E isolates were identical to the reference strains used.

In establishing the evolution rates of the Kenyan HCoVs, Tajima’s test showed an overall value of Tajima’s D statistic for the eight HCoV-OC43 pol gene segments as −1.31009 with p > 0.10 at 95 % CI. For HCoV-NL63, the overall value of Tajima’s D statistic for the 5 sequences was −0.97256 with p > 0.10 at 95 % CI. The evolutionary rates of the Kenyan HCoVs were also analyzed using Fu and Li’s D as well as Fu and Li’s F tests as these can accommodate small sample sizes. The overall Fu and Li’s D value for the HCoV-OC43 pol gene segments was −1.40980 and −0.97256 for HCoV-NL63, p > 0.10. The overall Fu and Li’s F statistic was −1.51361 for HCoV-OC43 and −0.95440 for HCoV-NL63, p > 0.10.

## Discussion

HCoVs have a global distribution and are endemic to many countries [5, 8, 23]. However, in Africa and especially sub-Saharan Africa, there is a dearth of information about these viruses in regard to their circulation dynamics, diversity, molecular characteristics and epidemiology [24].

Four out of the six known human coronavirus species were detected by RT-PCR assay, which has been shown to detect respiratory viruses with high sensitivity and specificity [25]. This finding echoes previous studies in Kenya [26] and globally where HCoVs were identified as significant etiological agents of ILI and acute respiratory illnesses (ARI) [9, 23]. HCoV-OC43 was the most commonly observed HCoV and this agrees with many previous studies that have reported HCoV-OC43 predominance [27]. HCoV-NL63 contributed to 2 % of ILI cases in our study period. This finding echoes the findings in South Africa by Smuts, [28] where they showed that HCoV-NL63, contributed to 2.4 % of ILI cases in the period of one and a half years. HCoV-229E contributed to 1 % of ILI cases in the study period, echoing results from other studies worldwide that show low detection of this HCoV [27]. This is comparable to what has been observed globally, where HCoV activity is sporadic throughout the year with no obvious distinct seasonality [29]. Overall, the symptoms associated with coronavirus infection were similar to what has been reported elsewhere [1, 7, 24] and it was difficult to distinguish an infection involving HCoV using symptoms alone. This justifies the WHO classification of respiratory viral infections into influenza-like-illnesses without clear distinction of a specific respiratory virus infection based on symptoms alone.

Out of 35 PCR-positive cases for HCoV, only 12 (34 %) yielded virus in LLCMK2 [30] and were confirmed by PCR. This was expected because molecular assays including PCR are more sensitive and specific at detecting pathogens [31, 32] than cell culture or serology. The low isolation rate may be attributed to low viral loads in the patients or the presence of inactivated viruses, potentially due to poor specimen handling and/or storage. Nonetheless, this finding agrees with reports of poor growth and a lack of cytopathic effect in cell cultures by HCoVs [33].

Phylogenetic analysis confirmed that the Kenyan isolates belonged to the *alpha* and *betacoronavirus* groups [6]. The Kenyan isolates clustered together with reference strains indicating that the Kenyan HCoVs are strain variants of those circulating elsewhere. Natural selection analyses revealed that the Kenyan HCoV-OC43 and HCoV-NL63 viruses were under negative selection, albeit not statistically significantly so. We did not detect any MERS-CoV or SARS-CoV.

This study had several shortcomings. The small sample size made it difficult to analyze evolutionary rates using the robust SLAC and FEL methods. Only a portion of the virus genome was analyzed and the evolution rates based on this small portion of the genome may be misleading. These shortcomings notwithstanding, it was demonstrated that four HCoV subtype strains were in circulation in Kenya from 2009 to 2012 and were similar to those circulating in other countries. However, sustained respiratory virus surveillance is necessary to monitor introductions of any of these dangerous species. Furthermore, full genome studies are required to provide comprehensive insight into the genetics and evolutionary characteristics of HCoVs in Kenya.

## Conclusions

There were yearly variations in the prevalence and circulation patterns of individual HCoVs in Kenya. This paper reports on the first molecular characterization of human Coronaviruses in Kenya, which play an important role in causing acute respiratory infections among children.
